# Evidence on Indications and Techniques to Increase the Future Liver Remnant in Children Undergoing Extended Hepatectomy: A Systematic Review and Meta-Analysis of Individual Patient Data

**DOI:** 10.3389/fped.2022.915642

**Published:** 2022-05-30

**Authors:** Juri Fuchs, Anastasia Murtha-Lemekhova, Lucas Rabaux-Eygasier, Markus Kessler, Fabian Ruping, Patrick Günther, Katrin Hoffmann

**Affiliations:** ^1^Department of General, Visceral and Transplantation Surgery, University of Heidelberg, Heidelberg, Germany; ^2^Generating Evidence for Diagnosis and Therapy of RarE LIVEr Disease: The RELIVE Initiative for Systematic Reviews and Meta-Analyses, University of Heidelberg, Heidelberg, Germany; ^3^Department of Pediatric Surgery, Hôpital Kremlin Bicêtre, Assistance Publique, Hôpitaux de Paris, Paris, France; ^4^Division of Pediatric Surgery, Department of General, Visceral and Transplantation Surgery, University of Heidelberg, Heidelberg, Germany

**Keywords:** pediatric surgery, ALPPS, portal vein embolization, pediatric liver tumors, pediatric liver resection, pediatric liver surgery

## Abstract

**Background:**

Techniques to increase the future liver remnant (FLR) have fundamentally changed the indications and criteria of resectability in adult liver surgery. In pediatric patients however, these procedures have rarely been applied and the potential benefit or harm as well as suited indications are unclear.

**Methods:**

A systematic literature search of MEDLINE, Web of Science, and CENTRAL was conducted. Based on a PRISMA-compliant, predefined methodology, all studies reporting pediatric patients (< 18y) undergoing liver resection with either associating liver partition and portal vein ligation for stages hepatectomy (ALPPS) or preoperative portal vein embolization or ligation (PVE/PVL) were included. Baseline data, periinterventional morbidity, increase of FLR and outcomes were analyzed.

**Results:**

15 studies reporting on 21 pediatric patients with a mean age of 4 years and 7 months (range 1.8 months – 17 years) were included. 12 ALPPS procedures, 8 PVE and 1 PVL were performed. The applied criteria for performing ALPPS or PVE were heterogenous and thresholds for minimally acceptable FLR varied. Mean FLR [% of total liver volume] before the intervention was 23.6% (range 15.0 – 39.3%) in the ALPPS group and 31.4% (range 21.5 – 56.0%) in the PVE group. Mean increase of FLR before stage 2 resection was 69.4% (range 19.0 – 103.8%) for ALPPS and 62.8% (range 25.0 – 108.0%) after PVE. No postoperative death occurred, one early intrahepatic recurrence after an ALPPS procedure was reported. Overall postoperative morbidity was 23.8%.

**Conclusion:**

Validated criteria for minimal FLR in pediatric liver resection are lacking and so are clear indications for ALPPS or PVE. In special cases, ALPPS and PVE can be valuable techniques to achieve complete resection of pediatric liver tumors. However, more data are needed, and future studies should focus on a definition and validation of posthepatectomy liver failure as well as the minimally needed FLR in pediatric patients undergoing extended hepatectomy.

**Systematic Review Registration:**

[www.clinicaltrials.gov], identifier [PROSPERO 2021 CRD42021274848].

## Introduction

Complete surgical resection is the mainstay in the therapy of most primary liver tumors in children (1). A fundamental paradigm in all liver resections is that enough functional liver parenchyma must remain after a partial hepatectomy in order to guarantee sufficient postoperative liver function (2). Posthepatectomy liver failure (PHLF) and small for size syndrome are well defined in the adult population, and have been shown to be a life-threatening complication (2–4). However, its role is less prominent in pediatric liver surgery, and definitions and prognostic values have not been validated in children (5). This has several reasons, some of which are evident, while others might not have been fully understood yet. Among the possible explanations is that the vast majority of malignant liver tumors in children respond to neoadjuvant chemotherapy and are accessible to standard liver resections without excessive postoperative risks (1). As a consequence, extended liver resection might simply be less often performed in children than in adults, which in turn reduces the risk of PHLF. Moreover, treatment protocols of pediatric liver tumors indicate liver transplantation for extensive tumors (6). Children are usually more likely to be listed for transplantation and receive grafts compared to adult oncologic patients. This avoids the necessity of extreme resections with minimal liver remnant to remove the tumor, which may be attempted more often in adult patients not eligible for liver transplant where alternatives are missing. Lastly, the percentage of liver tumors in adults developing in the context of chronic liver disease is much higher than in children (1). The compromised liver function before surgery increases the risk of PHLF (3). In addition, other comorbidities of the adult population, such as diabetes and obesity, have been linked with an increased risk of PHLF (7).

However, there are pediatric liver tumors with insufficient response to chemotherapy or large benign tumors with local complications, necessitating extended surgery for complete resection. While liver transplantation is the only option in some patients, it might be contraindicated or not available in certain cases. Moreover, for some patients, extreme resection with minimal liver remnant might be an alternative to avoid life-long immunosuppression (8–10). In these situations, however, the risk of PHLF might also be increased in children and is potentially life-threatening. In the adult population, PHLF has been clearly defined and different grades of this complications can be diagnosed in a reliable manner (4). Moreover, this allowed for the definition of cutoff values for minimal FLR, that have been extensively investigated and defined for adult patients (2, 11). In the pediatric population, such definitions are lacking, and there is no consensus on a validated definition of post-resection liver insufficiency. While some authors applied methods from adult liver surgery (5, 12, 13), it is unknown whether these principles derived from research and experience in adult liver resection can be transferred to pediatric hepatobiliary surgery.

In cases of insufficient FLR, techniques to increase the future liver remnant (FLR) have long been introduced for adults and increased the indications for liver resections with remarkable success. Notably, portal vein embolization (PVE) or portal vein ligation (PVL), and more recently, associating liver partition and portal vein ligation for staged hepatectomy (ALPPS) have been introduced, adopted, and now been extensively investigated. They are routinely applied in many hepatobiliary centers around the world and fundamentally changed the criteria of resectability. In the pediatric population, however, these procedures have only rarely been applied and reported. Virtually, only a few case reports of PVE, PVL and ALPPS in children have been published.

Aim of this systematic review with meta-analysis of individual patient data is to investigate possible indications, safety, efficacy and outcome of techniques to increase the future liver remnant in pediatric patients undergoing extended hepatic resection.

## Materials and Methods

### Ethics

All included studies stated consent for publication of the patient data by either approval from the relevant committees or the parents. As only anonymized, previously published data were part of our analyses, the institutional review board of the Medical Faculty of the University of Heidelberg approved the data collection and conduct of the present study without additional consent being necessary (Signed July 2013 and Section 15, paragraph 1 of the code of medical ethics of the federal state of Ba-den-Wuerttemberg, Germany).

### Review Structure and Search Strategy

The authors of this review have developed a methodologic structure for systematic reviews with the specific aim of generating evidence for rare diseases, which is the basis of this study. It is part of the *RELIVE* research initiative (Generating evidence for RarE LIVEr Disease). Systematic reviews of these rare diseases are often based on analyses of individual patient data, as it has been performed in the present study.

This review is conducted in accordance with the *Preferred Reporting Items for Systematic Reviews and Meta-Analyses (PRISMA)* guidelines and was registered with the *Prospective Register of Systematic Reviews* (PROSPERO 2021 CRD42021274848) before starting the analysis.

The search strategy used for this systematic review follows the recommendation of the Study Center of the German Society of Surgery (14). A combination of the following free text and medical subject heading (MeSH) terms were used for the systematic literature search: ALPPS, associating liver partition, *in situ* split, portal vein embolization, portal vein ligation, liver failure, small for size, hepatoblastoma, children, pediatric, future liver remnant. Three different databases (MEDLINE via PubMed, Web of Science, and CENTRAL) were explored. The exact search algorithms are provided in the Supplementary Material (file 1). In addition, references of the relevant studies were screened for eligible articles. Moreover, data on pediatric ALPPS procedures were requested from the international ALPPS registry. The last search was performed on February 10th 2022.

### Study Selection Criteria and Selection Process

No study types were excluded from this systematic review with the following inclusion criteria defined:

• patient age < 18 years and,
• Associating liver partition and portal vein ligation performed or,
• portal vein ligation with second stage liver resection performed or,
• portal vein embolization with second stage liver resection performed.

All studies that were found by the systematic search were screened for eligibility by two reviewers independently (JF and AML). In a first phase, the abstracts were analyzed, and unsuitable articles were excluded. In the second phase, all eligible studies based on the abstract screening were reviewed based on the full texts and decision on in- or exclusion was made. Dissent between the two reviewers was resolved after consulting with a third reviewer (KH).

### Data Extraction and Investigated Variables

Based on a predefined and standardized form, data were extracted by the two reviewers independently. All accessible data items were included in this form. The complete list of all extracted variables is added as Supplementary Material (file 2).

### Risk of Bias Assessment

The investigated procedures have only rarely been applied and reported in children. Thus, predominantly case reports and small case series were expected to be found and included in this study. The risk of bias assessment tool by Murad et al. that has been specifically developed for this kind of studies, was applied for evaluating the quality and risk of bias of the included reports (15).

### Statistical Analyses and Certainty of Evidence

Statistical analyses were performed with R (version 3.6.2) (16). Data was entered individually, and descriptive analyses performed. For continuous data, means, medians, standard deviations (SD) or ranges were calculated. For categorial data, numbers with percentages are given. Significances of the differences between means were tested with the Wilcoxon-Mann-Whitney-U-Test at a level of significance of 95%. The GRADE criteria were applied for determining the certainty of evidence and strength of recommendations (17).

### Applied Terms and Definitions

#### Associating Liver Partition and Portal Vein Ligation

Associating liver resection and portal vein ligation for staged hepatectomy (ALPPS) was defined for this systematic review as all kind of completed two stage hepatectomy with the first stage consisting of parenchymal transection along the definitive resection line along with ligation of the portal vein branch(es) supplying the resected segments. All variations of ALPPS, that has been first describe by Schnitzbauer et al. (18), such as laparoscopic first stage or monosegment ALPPS (19) were included in our study.

#### Portal Vein Embolization

Portal vein embolization (PVE) was defined as radiological, percutaneous transhepatic embolization of the portal vein branch that supplies the liver lobe that is resected in a following liver resection (20, 21).

#### Portal Vein Ligation

Portal vein ligation (PVL) was defined as two stage hepatectomy with the first stage being a surgical ligation of the portal vein branch supplying the liver segments that are to be resected in the second stage (20, 22).

#### Posthepatectomy Liver Failure

The definition by the International Study Group of Liver Surgery (ISGLS) for PHLF was applied in our study to evaluate the occurrence of hepatic insufficiency after liver resection (4).

#### Clavien-Dindo Classification

Postoperative complications were graded according to the validated Clavien-Dindo classification of postoperative surgical complications (23).

#### PRETEXT Staging

For all intrahepatic tumors, the pretreatment extent of disease (PRETEXT) staging system was applied (24). Developed and validated for hepatoblastoma (HB) in children, this system allows for a comprehensible, surgery-directed, and standardized staging of the local extension of liver tumors in pediatric patients.

## Results

### Literature Search and Study Selection

**FIGURE 1:**
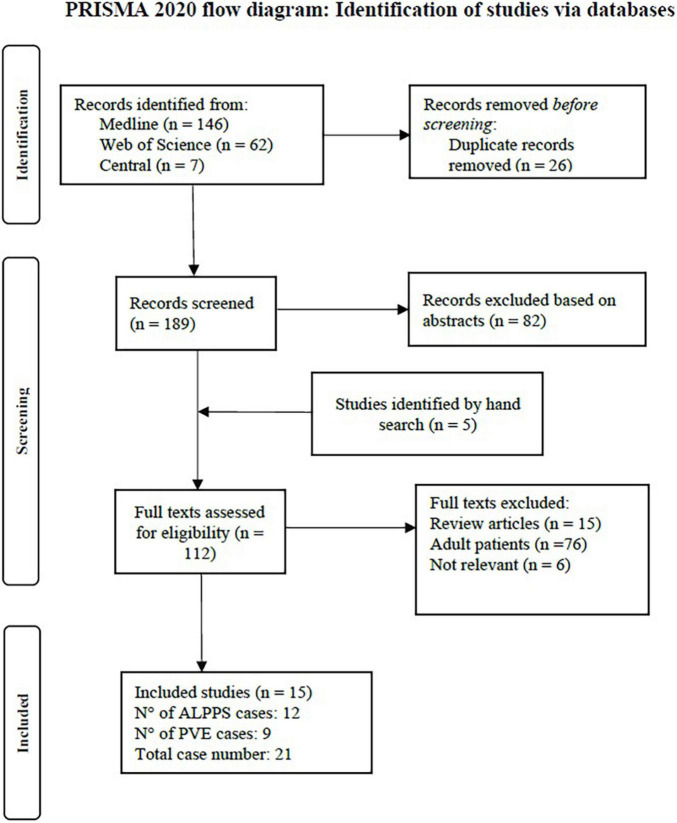
PRISMA flow chart of the study selection and inclusion process.

The PRISMA flow diagram (Figure 1) shows the study selection process. 194 studies were screened for eligibility (189 retrieved by the systematic literature search and 5 by free hand search). 15 studies met inclusion criteria and are discussed and analyzed in this systematic review. These studies report on 21 pediatric patients, of which 12 underwent ALPPS procedures, 8 PVE and 1 PVL.

### Critical Appraisal of Included Studies and Risk of Bias

As anticipated when drafting the protocol for this systematic review, only few studies on the investigated interventions were found, and the number of pediatric cases hitherto reported is very low. Consequently, no large cohort studies or comparative trials were found. Mostly individual case reports or small case series on ALPPS or PVE in children have been published so far and were included in our study. This implies that selection bias was high in most of these reports. Only two studies presented small series of all patients treated with ALPPS or PVE in one institution during a given period (2, 25, 6), and therefore had a lower selection bias. While the low number of cases involves several biases, it also implied that the individual cases were presented in detail and many factors were reported in the included studies. The length of follow up differed substantially across the studies and was not long enough to allow for an appropriate evaluation of oncologic outcome in some studies. The quality of describing the detailed algorithm or clinical circumstances, that led to the decision to perform ALPPS or PVE, differed across the studies and was insufficient in some. Detailed postoperative laboratory values that would allow for evaluation of PHLF according to the ISGLS definition (4) were mostly incomplete. Overall, most studies provided acceptable quality of pre- and postoperative data, varying intraoperative details, and acceptable length of follow up. The details of the risk of bias assessment according to the tool by Murad et al. can be found in Table 1 (***all tables at the end of the manuscript***).

**TABLE 1:** Risk of bias assessment of included studies.

Study	Selection	Ascertainment	Causality	Reporting	Overall ROB
(27)	high	low	low	low	Low
(28)	high	low	moderate	moderate	moderate
(29)	high	moderate	low	low	moderate
(30)	high	low	moderate	moderate	moderate
(31)	high	moderate	low	moderate	moderate
(32)	high	low	low	moderate	moderate
(33)	high	low	low	moderate	Low
(34)	high	low	moderate	low	moderate
(35)	high	low	low	low	Low
(36)	high	low	low	low	Low
(37)	high	moderate	low	low	moderate
(38)	high	low	low	low	Low
(25)	low	moderate	high	low	moderate
(26)	low	low	high	low	moderate
(39)	high	low	moderate	low	moderate

### Patient Baseline Data

In total, 21 patients were included in this analysis. Twelve patients underwent an ALPPS procedure, 8 patients PVE and 1 patient PVL. For exploratory analyses and comparisons, patients were grouped into an ALPPS group (*n* = 12) and a PVE/PVL group (*n* = 9 patients). Mean age at the time of intervention was 4 years and 7 months (ALPPS: 5 years and 2 months; PVE/PVL: 3 years and 11 months). The mean age of the two groups did not differ significantly (*p* = *0.741*). Hepatoblastoma was the most common diagnosis with 12 of 21 cases (7/12 in ALPPS and 5/9 in PVE/PVL). Other malign liver tumors were diagnosed in 4 patients (3/12 ALPPS, 1/9 PVE/PVL) and benign liver tumors in 3 children (2/12 in ALPPS, 1/9 in PVE/PVL). One complex bile duct injury with necessity of liver resection and biliary reconstruction and one inflammatory pseudotumor in the hepatic hilum that necessitated extended liver resection were reported (both PVE/PVL group). In the 19 cases with intrahepatic tumors, 18 were PRETEXT III and one PRETEXT IV (PRETEXT III: 11/12 in ALPPS, 7/9 in PVE/PVL; 1/12 PRETEXT IV in ALPPS). See Tables 2, 3 for an overview of all patients included in this study. Tables 4, 5 show syntheses of the patient data.

**TABLE 2:** Overview of pediatric patients undergoing ALPPS.

Patient no°	References	Patient age and weight	Diagnosis	Affected segments (PRETEXT)	FLR before ALPPS stage 1	Timing & FLR ALPPS stage 2	Increase of FLR	Type of resection	Postoperative complications	Follow up
1	Chan et al. (28)	6y, 16.9 kg	HB	IV, V, VIII PRETEXT III	21.2%	Day 8 30.2%	42.2%	Right trisectionectomy	No complications Discharged day 16	NED at 2m
2	Wiederkehr et al. (25)	12y 4m, 49 kg	RMS	IV, VII, VIII PRETEXT III	25.5%	41%	60.7%	Right trisectionectomy	No complications	Not available
3	Wiederkehr et al. (25)	4y, 23 kg	HCC	I, II, III, IV, V, VIII PRETEXT III	39.3%	74%	88.3%	Left trisectionectomy	No complications	Not available
4	Wiederkehr et al. (25)	10y 3m, 34 kg	FNH	IV, VIII PRETEXT III	21.7%	40%	84.3%	Right trisectionectomy	No complications	Not available
5	Wiederkehr et al. (25)	17y 3 m, 43 kg	HB	IV, V, VI, VII, VIII PRETEXT III	14.7%	17.5%	19.0%	Right trisectionectomy	No complications	Not available
6	Wiederkehr et al. (25)	3y 2 m, 13 kg	HB	IV, V, VIII PRETEXT III	26.5%	Day 11 54%	103.8%	Right trisectionectomy	No complications	Not available
7	Sidorov et al. (36)	2y 5 m	Embryonal Sarcoma	IV, V, VII, VIII PRETEXT III	15.0%	Day 8 40.0%	75.7%	Right trisectionectomy	No complications Discharged day 15	NED at 11m
8	Qazi et al. (34)	10 m 8.6 kg	HB	IV, V, VI, VIII PRETEXT III	30.0%	Day 15 48.3%	61.0%	Right trisectionectomy	Resp. infection (II), discharged day 18	DRD at 1.5m
9	Hong et al. (30)	1.8 m 4.4 kg	HB	IV, V, VI, VII, VIII PRETEXT III	24.7%	Day 8 38.0%	91.0%	Right trisectionectomy	Ileus, PHLF (II) Discharged day 15	Not available
10	Xu et al. (39)	9 m, 10 kg	Hamartoma	IV, V, VI, VII, VIII PRETEXT III	22.7%	Day 8 40.0%	75.7%	Right trisectionectomy	No complications Discharged day 24	NED at 2m
11	Figueroa et al. (35)	3y	HB	I, II, III, IV, V, VII,VIII PRETEXT IV	21.3%	Day 16 32.6%	52.6%	Monosegment IV ALPPS	Pneumonia (II) Discharged day 30	NED at 55m
12	Akhaladze et al. (40)	20 m	HB	IV, V, VII, VIII PRETEXT III	20.5%	Day 6 33.0%	61.0%	Right trisectionectomy	No complications Discharged day 14	NED at 15m

*Abbreviations: ALPPS: Associating liver partition and portal vein ligation, DRD: Disease related death, FLR: Future liver remnant, NED: No evidence of disease, PVL: Portal vein ligation, PVE: Portal vein embolization, HB: Hepatoblastoma, HCC: Hepatocellular carcinoma, FNH: Focal nodular hyperplasia, RMS: Rhabdomyosarcoma, PRETEXT: Pretreatment extent of disease.*

**TABLE 3:** Overview of pediatric patients undergoing PVE.

Patient no°	References	Patient age and weight	Diagnosis	Affected segments (PRETEXT)	FLR before PVE	Timing & FLR at resection	Increase of FLR	Type of resection	Postoperative complications	Follow up
13	Kaneko et al. (31)	8y 11m	Inflammatory pseudotumor	Porta hepatis including portal vein bifurcation	29.3%	Day 30 46.1%	57.3%	Right trisectionectomy	No complications Discharged day 7	NED at 14m
14 ^†^	Tanaka et al. (37)	13y	HCC	I, II, III, IV, V, VIII PRETEXT III	56.0%	Day 10 70.6%	26.0%	Left trisectionectomy	Bile leakage (III) Discharged day 16	NED at 24m
15	Terraz et al. (38)	14m, 6.8 kg	Mesenchymal Hamartoma	IV, V, VI, VIII PRETEXT III	21.5%	Day 35 42.6%	108.0%	Right trisectionectomy	No complications Discharged day 12	NED at 48m
16	Glinka et al. (29)	5y 5m, 17.1 kg	Iatrogenic bile duct injury (Strasberg E4)	Injury of biliary confluence and right hepatic artery	34.0%	Day 30 52.5%	40.8%	Right hemi-hepatectomy	No complications Discharged day 10	NED/No symptoms at 24m
17	Le at al. (33)	14m	HB	IV, V, VI PRETEXT III	28.0%	Day 35 35.0%	25.0%	Right trisectionectomy	No complications Discharged day 24	NED at 15m
18	Kannappan et al. (32)	18m	HB	IV, V, VI, VIII PRETEXT III	26.8%	Day 25 50.0%	104.0%	Right trisectionectomy	Bile leakage (III) Discharged day 7	NED at 24m
19	Wildhaber et al. (26)	14m 7.6 kg	HB	PRETEXT III	1.5% of body weight	4-6 weeks 2.7%	76.3%	Right trisectionectomy	No complications	Not available
20	Wildhaber et al. (26)	26m 11.5 kg	HB	PRETEXT III	1.0% of body weight	4-6 weeks 1.4%	33.3%	Right trisectionectomy	No complications	Not available
21	Wildhaber et al. (26)	7m 6.9 kg	HB	PRETEXT III	1.0% of body weight	4-6 weeks 1.7%	72.2%	Right trisectionectomy	No complications	Not available

*† Portal vein ligation performed; Abbreviations: ALPPS: Associating liver partition and portal vein ligation, FLR: Future liver remnant, NED: No evidence of disease, PVL: Portal vein ligation, PVE: Portal vein embolization HB: Hepatoblastoma, HCC: Hepatocellular carcinoma, PRETEXT: Pretreatment extent of disease.*

**TABLE 4:** Patient characteristics and data on FLR.

	ALPPS (*n* = 12)	PVE/PVL (*n* = 9)	All patients (*n* = 21)
Mean age	5y 1m (range 1.8-207 m)	3y 11 m (range 7 -156 m)	4y 7m (range 1.8-207 m)
Mean FLR/TLV before intervention	23.6% (range 15.0-26.5%)	31.4% (range 21.5-56.0%)	27.0% (range 15.0-56.0%)
Median time between stage 1/PVE and stage 2/resection	11 days (range 8-16 days)	30 days (range 10-42 days)	11 days (range 8-42 days)
Mean FLR/TLV before stage 2	40.7% (range 17.5-74.0%)	46.9% (range 35.0-70.6%)	43.6% (range 17.5-74.0%)
Mean increase of FLR	69.4% (range 19.0-103.8%)	62.8% (range 25.0-108.0%)	65.5% (range 19.0-108.0)
Mean length of hospital stay^†^	14.8 days (range 6-30 days)	11.2 days (range 6-24 days)	13.5 (range 6-30 days)
Overall postoperative morbidity	3 patients with complications^‡^ (4x Clavien-Dindo Grade II)	2 patients with complications (2x Clavien-Dindo Grade III)	5 patients with complications
Recurrence	1 patient	/	1 patient

*† Days after liver resection; ‡One patient suffered from 2 complications; Abbreviations: ALPPS: Associating liver partition and portal vein ligation, FLR: Future liver remnant, PVL: Portal vein ligation, PVE: Portal vein embolization, TLV: Total liver volume.*

**TABLE 5:** Details on the types of liver tumors or underlying disease.

	ALPPS (*n* = 12)	PVE (*n* = 9)	All patients (*n* = 21)
Hepatoblastoma	7	5	12
HCC	1	1	2
Embryonal sarcoma	1	−	1
Rhabdomyosarcoma	1	−	1
Mesenchymal hamartoma	1	1	2
Focal nodular hyperplasia	1	−	1
Inflammatory pseudotumor	−	1	1
Bile duct injury	−	1	1
PRETEXT III	11	7	18
PRETEXT IV	1	−	1

*Abbreviations: HCC: Hepatocellular carcinoma, PRETEXT: Pretreatment extent of disease, ALPPS: Associating liver partition and portal vein ligation, PVL: Portal vein ligation, PVE: Portal vein embolization.*

### Applied Criteria and Reasons for Performing Associating Liver Resection and Portal Vein Ligation for Staged Hepatectomy or Portal Vein Embolization / Portal Vein Ligation

Except for one case, all reports stated potentially insufficient FLR as the main reason for performing an ALPPS procedure or a PVE. However, the detailed description or definition of the applied criteria were mostly lacking. Different limits or threshold values for FLR were applied across the studies. The range of the defined limit as FLR was <15% to <40% and was almost exclusively based on the experience in adult liver resection or liver transplantation. In three cases, the liver transplantation was considered indicated, given the local extent of the liver tumors, but was not available or contraindicated in these patients. Thus, ALPPS or PVE were applied in these cases and extreme resections were performed as last attempt to cure these children. See Table 6 for details.

**TABLE 6:** Applied criteria and reason for performing ALPPS or PVE/PVL.

Applied criteria for performing ALPPS/PVE	ALPPS (*n* = 12)	PVE (*n* = 9)	All patients (*n* = 21)
FLR/TLV < 15%	1	0	1
FLR/TLV < 25%	5	0	5
FLR/TLV < 30%	1	3	4
FLR/TLV < 40%	5	0	5
FLR < 1.5% of body weight	0	5	5
Not mentioned/other	0	1	1
Liver transplantation not available or contraindicated	2	1	3

*ALPPS: Associating liver partition and portal vein ligation, FLR: Future liver remnant, PVL: Portal veinxs ligation, PVE: Portal vein embolization, TLV: Total liver volume.*

### Future Liver Remnant Before Interventions

Mean FLR (in% of total liver volume (TLV)) before ALPPS stage 1 or PVE/PVL was 23.6% in the ALPPS group (range 15.0 – 26.5%) and 31.4% in the PVE/PVL group (range 21.5 – 56.0%), the difference being not significant between the ALPPS and PVE/PLV group (*p* = 0.168).

### Associating Liver Resection and Portal Vein Ligation for Staged Hepatectomy Procedures

Of the 12 ALPPS procedures, 10 were right trisectionectomies. One left trisectionectomy and one mono-ALPPS procedure were performed. The median time between stage 1 and stage 2 was 11 days (range 8 – 16 days). The mean increase of FLR before stage 2 was 69.4% (range 19.0 – 103.4%). The increase of FLR before stage 1 to stage 2 of ALPPS was significant (*p* = *0.011)*. No major intraoperative hemorrhage was reported. Three patients suffered from four postoperative complications. All four complications were Clavien-Dindo grade II and not liver-specific. PHLF grade A was reported in one patient. The mean length of hospital stay was 14.8 days (range 6 – 30 days). See Tables 2, 4.

### Portal Vein Embolization / Portal Vein Ligation Procedures

Of the 9 resections, 7 were right trisectionectomies, one left trisectionectomy and one right hemihepatectomy. The median time between portal vein embolization and resection was 30 days (range 10 – 42 days). The mean increase of FLR before resection was 62.8% (range 25.0 – 108.0%). The difference between FLR before PVE/PVL and before liver resection not was significant (*p* = *0.430)*. Two patients suffered from bile leakage (Clavien-Dindo grade III) and needed re-intervention. In both cases, the complication was treated successfully and both patients were free of disease and in good health at two years follow up. No case of PHLF was reported. The mean length of hospital stay was 11.2 days (range 6 – 24 days). See Tables 3, 4.

The mean increases of FLR did not differ significantly between the ALPPS group and the PVE group (*p* = *0.674).*

#### Follow up

Follow up was available in 6 of 12 patients in the ALPPS group and 6 of 9 in the PVE/PLV group. One early recurrence of HB in the liver remnant, one month after resection was reported in the ALPPS group. All other patients with follow up data available were free of disease. Median follow up was 15 months (range 1.5 – 55 months). In those patients with information on liver function available, no symptoms or laboratory parameters indicated long term sequelae after the extended resections.

## Discussion

Extended liver resections in children are rare procedures. Portal vein embolization and ALPPS substantially enhanced the surgical options for adult patients with extensive liver tumors and have been well investigated. Their relevance and applicability in the pediatric population is unclear, however. This is the first systematic review investigating techniques to increase the future liver remnant in pediatric patients undergoing liver resection. Clear and validated definitions of PHLF in children are lacking, and validated limits for FLR have not been established yet. The pathomechanism and risk factors for PHLF in pediatric patients are poorly understood. As a consequence, insights and innovations derived from research or clinical practice in adult liver surgery are often transferred into pediatric hepatobiliary surgery. However, this bears the risk of neglecting important differences between adult and pediatric patients. For example, Needham et al. showed that children returned sooner than adults to normal liver function test after major hepatectomy (41). Yet, profound understanding of physiologic differences in liver regeneration between children and adults is lacking.

### Indications of Associating Liver Resection and Portal Vein Ligation for Staged Hepatectomy and Portal Vein Embolization in Children

While primary hepatic tumors are by far the most common conditions necessitating extended liver resections in children, other rare diseases can be indications for major hepatectomy. In the adult population, ALPPS or PVE are usually considered in a situation where the estimated FLR is below 25% of the total liver volume (TLV) (3, 11). This lower limit might be set higher for patients with pre-existing liver disease or dysfunction (3). In our study, 12 of 21 patients (4 of 12 patients in the ALPPS group and 8 of 9 patients in the PVE group) had an FLR >25% before starting the two-stage procedures. Except for neoadjuvant chemotherapy, no chronic liver diseases were present in the 21 patients and compromised liver function before surgery was not reported in any of the patients. Whether ALPPS and PVE were indeed necessary and indicated in these cases, or if conventional trisectionectomy would have been safe, remains questionable. The lack of validated definitions of PHLF and limits for minimal FLR in pediatric liver resection represents a major problem in the evaluation of indications for ALPPS and PVE in children. This important issue is discussed in a separate section of the discussion. In three cases, ALPPS/PVE were applied in children that were usually regarded as being candidates for total hepatectomy and liver transplantation due to extensive liver tumors. One of these patients had contraindications and the other two were treated in countries with a medical system without availability of liver transplantation. For those children, ALPPS or PVE probably offered the only chance of cure with extreme resection (mono-ALPPS in one case). In these situations, ALPPS or PVE might be indicated in pediatric patients even in the face of insufficient evidence on the benefit and outcomes in children. They should at least be considered as possible treatment options for these special cases.

### Efficacy of Associating Liver Resection and Portal Vein Ligation for Staged Hepatectomy and Portal Vein Embolization on the Increase of FLR in Children

**FIGURE 2:**
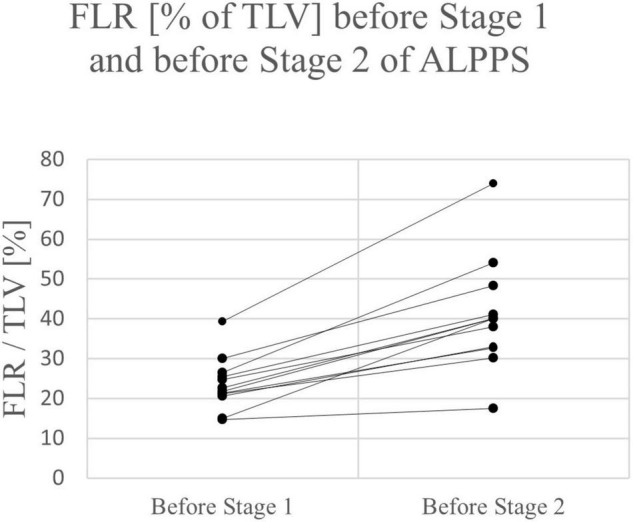
FLR of individual patients before stage 1 and before stage 2 of ALPPS.

**FIGURE 3:**
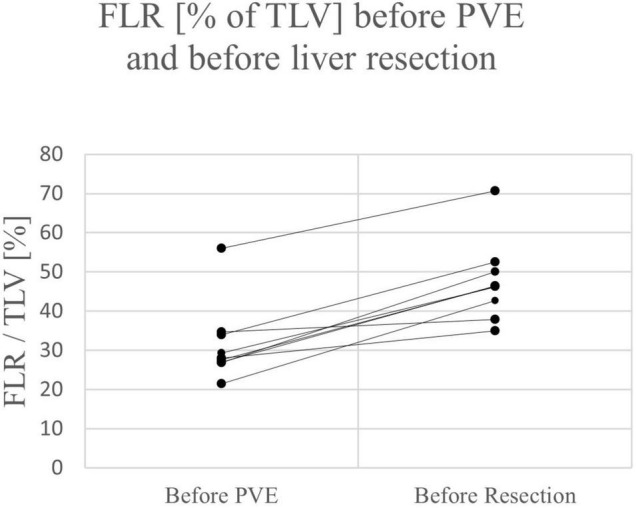
FLR of individual patients before PVE and before liver resection.

**FIGURE 4:**
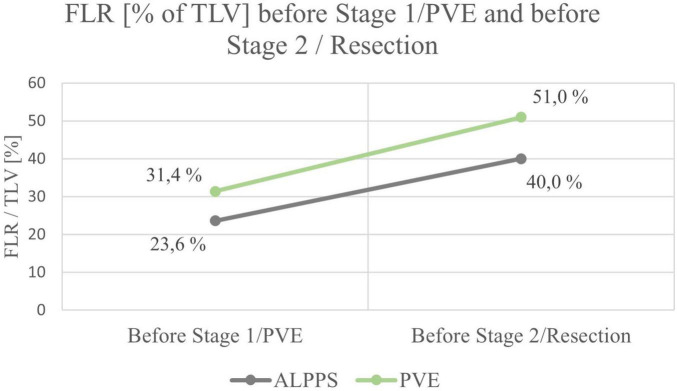
Mean FLR before and after the first stage of the interventions.

Except for one patient in the ALPPS group, a relevant increase of FLR was observed after the first stage of ALPPS or PVE in all children (range 25 – 104%) (see Figures 2, 3). All patients had an FLR > 30% before the liver resection. These results show that the fundamental idea of these procedures seems to be applicable in pediatric patients and the findings suggest that a considerable increase of FLR can be reliably achieved with ALPPS and PVE. The increase of FLR was significant in the ALPPS group (69.4%), but not-significant in the PVE/PVL group (62.8%) (see Figure 4). A stronger increase of FLR achieved by ALPPS compared to PVE has been shown in adult liver surgery (42). However, the findings of our study might be biased by the low number of patients and further reporting and research is needed to investigate the effectivity of ALPPS vs. PVE in children.

### PHLF and Minimal FLR in Children Undergoing Major Liver Resection

The lack of validated definitions of PHLF, and, as a consequence, the lack of evidence-based recommendations for minimally acceptable FLR in children is reflected by the wide range of applied criteria for performing ALPPS or PVE among the different reports. While FLR > 25% is usually regarded as being sufficient in adult patients (11), our study showed that the applied limits in children varied between 15 and 40% in the included studies of ALPPS or PVE. There is only one study on pediatric patients undergoing major hepatectomy with a focus on FLR and occurrence of PHLF (43). The study by Akhaladze et al. could not produce sufficient evidence to have clear guidelines. However, first evidence suggests that FLR < 25% might still be safe in children undergoing extended liver resection (43). An evidence base for applying ALPPS or PVE in children is missing and so are clear indications for these procedures.

In order to perform comparable analyses and produce reliable evidence on limits for FLR in children, a standardized definition of PHLF must be applied, also in the pediatric population. There are very few studies that specifically reported or investigated PHLF in pediatric patients. Hirata et al. reported on four patients that suffered from PHLF after trisectionectomy with consecutive necessity of liver transplantation (5). The authors defined PHLF as a peak of serum bilirubin >7.0 mg/dl at any postoperative day and did not differentiate between different grades of PHLF. In other studies, specific information on occurrence of PHLF after pediatric hepatectomy are often lacking. Some authors applied the internationally recognized ISGLS definition of PHLF in children (13, 43, 44). These criteria have been developed for and validated in adult populations only (4). As a result, reliable information on rates of PHLF in pediatric patients, in particular milder grades, are largely unknown. In the few studies that reported on PHLF after pediatric partial hepatectomy, rates of PHLF range from 0 to 17% (13, 43, 44). Also, in the included reports on ALPPS and PVE in children, detailed postoperative data, including serum bilirubin and international normalized ratio, as well as information on ascites, were insufficient. Only in one report, ISGLS grade A PHLF could be diagnosed based on the reported data. However, it can be concluded that severe PHLF was not observed after any of the reported resections, even when performing mono-segment ALPPS.

### Safety of Associating Liver Resection and Portal Vein Ligation for Staged Hepatectomy and Portal Vein Embolization in Children

Three patients in the ALPPS group and two patients in the PVE group suffered from postoperative complications. No postoperative death occurred. No complications were reported after the first step of ALPPS or during/after PVE. Across all 21 patients, overall morbidity was 24%, major complications (≥ Clavien-Dindo Grade III) occurred in two patients (10%). In literature, reported morbidity after pediatric liver resection ranges from 16 to 69%, averaging at about 30% across different studies (13). Given the high-risk nature of extended liver resection, the outcome after ALPPS and PVE resections seems acceptable. Follow up data was insufficient in most of the included studies to allow for an analysis of the oncologic and long-term outcome of pediatric patients undergoing ALPPS/PVE.

### Study Limitations and Strength of Recommendations

Given the rarity of ALPPS and PVE in pediatric patients, the body of literature is limited, which represents the major limitation of this study. Obviously, the low number of reported cases in the world literature and the heterogeneity of the included studies hampered the comparison and the statistical analyses. Moreover, the lack of standardized definitions of complications in pediatric liver surgery, such as PHLF, further introduced a risk of bias. In addition, long term follow up data was largely missing. As a consequence, this systematic review must be seen as an overview and data synthesis with the aim of generating research hypotheses and lying foundations for future studies. Major strength of this systematic review is the complete synthesis of all reported cases of pediatric ALPPS and PVE/PVL with detailed outcome data. We showed that the principles of ALPPS and PVE might be applicable in children and that a significant increase of FLR can be achieved. Thus, a data basis for the development of research questions and future studies is provided. The level of evidence derived from the included studies, that are mainly case series, is low, and the strength of recommendations is conditional.

## Conclusion

➢ ALPPS and PVE are technically feasible procedures in children.
➢ Significant increase of FLR can be achieved in most pediatric patients by ALPPS and PVE.
➢ First evidence suggests that they are not associated with excessive risks compared to one-stage extended liver resection or liver transplantation.
➢ Validated definitions of PHLF as well as evidence on minimally acceptable FLR in pediatric patients are lacking, and thus an evidence base for performing ALPPS and PVE in children.
➢ In pediatric PRETEXT IV liver tumors or in case of contra-indications or unavailability of liver transplantation, ALPPS or PVE may offer the chance of complete tumor resection as last possible surgical procedure and should be considered.
➢ Further studies are needed to investigate the role of PHLF and FLR in pediatric liver resections.
➢ The potential benefit of ALPPS and PVE in children has to be further clarified, for example by initiating an open access pediatric ALPPS/PVE registry.

## Reporting Checklist

This systematic review has been conducted in accordance with the PRISMA guidelines. The authors have completed the PRISMA reporting checklist for systematic reviews and meta-analyses.

## Data Availability Statement

The original contributions presented in the study are included in the article/Supplementary Material, further inquiries can be directed to the corresponding author/s.

## Author Contributions

JF, AM-L, LR-E, MK, FR, PG, and KH contributed to conception and design of the study. JF, AM-L, and KH conducted the systematic literature search, study selection, and extracted the data. JF and AM-L performed the statistical analysis. JF wrote the initial draft of the manuscript. AL, KH, and LR-E wrote sections of the manuscript. All authors substantially contributed to manuscript revision, read, and approved the submitted version.

## Conflict of Interest

The authors declare that the research was conducted in the absence of any commercial or financial relationships that could be construed as a potential conflict of interest.

## Publisher’s Note

All claims expressed in this article are solely those of the authors and do not necessarily represent those of their affiliated organizations, or those of the publisher, the editors and the reviewers. Any product that may be evaluated in this article, or claim that may be made by its manufacturer, is not guaranteed or endorsed by the publisher.

## Abbreviations

ALPPS: Associating liver resection and portal vein ligation for staged hepatectomy
FLR: Future liver remnant
HB: Hepatoblastoma
ISGLS: International Study Group of Liver Surgery
PHLF: Posthepatectomy liver failure
PVE: Portal vein embolization
PVL: Portal vein ligation
SD: Standard devition
TLV: Total liver volume.
