# Supplementary material for: Evidence on Indications and Techniques to Increase the Future Liver Remnant in Children Undergoing Extended Hepatectomy: A Systematic Review and Meta-Analysis of Individual Patient Data
Source: Front Pediatr. 2022 May 30;10:915642. doi: 10.3389/fped.2022.915642 (PMC9197416; doi:10.3389/fped.2022.915642)
Supplement: Supplementary file 2 [file Table_2.DOCX]

Applied search algorithms for a systematic review on pediatric ALPPS and PVE (Fuchs et al.)

1. Medline via Pubmed
2. ((liver[tiab] AND failure[tiab]) OR (small for size[tiab])) AND hepatoblastoma[tiab]
3. (ALPPS[tiab] OR "Associating liver partition"[tiab] OR "future liver remnant"[tiab] OR "portal vein embolization"[tiab]) AND (Children[tiab] OR pediatric[tiab] OR boy[tiab] OR girl[tiab] OR infant[tiab] OR toddler[tiab] OR adolescent[tiab] OR baby[tiab])
4. (Child OR pediatric* OR hepatoblastoma OR juvenile OR kid OR infant OR baby OR toddler) AND (ALPPS OR "Associating Liver Partition with Portal vein ligation for Staged hepatectomy" OR ISLT OR "in situ split" OR "portal vein ligation")
5. Web of Science

AB = (((ALPPS) OR ("Associatin liver partition") OR ("PVE") OR ("Portal vein embolication") OR ("Future liver remnant")) AND (Children OR pediatric OR infant OR toddler OR adolescent))

1. Central

((ALPPS):ti,ab,kw OR (Associating liver partition and portal vein ligation):ti,ab,kw OR (portal vein ligation):ti,ab,kw OR (portal vein embolizaion):ti,ab,kw) AND ((children):ti,ab,kw OR (pediatric):ti,ab,kw OR R (boy):ti,ab,kw OR (girl):ti,ab,kw OR (infant):ti,ab,kw OR (toddler):ti,ab,kw OR (adolescent):ti,ab,kw OR (baby):ti,ab,kw)
