# Supplementary material for: Evidence on Indications and Techniques to Increase the Future Liver Remnant in Children Undergoing Extended Hepatectomy: A Systematic Review and Meta-Analysis of Individual Patient Data
Source: Front Pediatr. 2022 May 30;10:915642. doi: 10.3389/fped.2022.915642 (PMC9197416; doi:10.3389/fped.2022.915642)
Supplement: Supplementary file 3 [file Table_3.DOCX]

Full list of investigated variable in the systematic review on ALPPS/PVE in children (Fuchs et al.)

Authors

Year of publication

Location/Country

Patient age

Gender

Symptoms before surgery

Body weight

Body height

Preoperative AFP

Preoperative imaging diagnostic

Affected segments

PRETEXT

Histologic diagnosis

Treatment before ALPPS or PVE

FLR/TLV before ALPPS/PVE

Operation time ALPPS stage 1

Blood loss stage 1

Complications stage 1/ PVE

FLR/TLV after first stage ALPPS/PVE

Increase of FLR [%]

Days between stage 1/PVE and resection

Type of resection

Blood loss stage 2/resection

Complications of stage 2/resection

PHLF

Length of hospital stay

Adjuvant treatment

Length of follow up

State at last follow up
